# Sage, *Salvia officinalis* L., Constituents, Hepatoprotective Activity, and Cytotoxicity Evaluations of the Essential Oils Obtained from Fresh and Differently Timed Dried Herbs: A Comparative Analysis

**DOI:** 10.3390/molecules26195757

**Published:** 2021-09-23

**Authors:** Hamdoon A. Mohammed, Hussein M. Eldeeb, Riaz A. Khan, Mohsen S. Al-Omar, Salman A. A. Mohammed, Mohammed S. M. Sajid, Mohamed S. A. Aly, Adel M. Ahmad, Ahmed A. H. Abdellatif, Safaa Yehia Eid, Mahmoud Zaki El-Readi

**Affiliations:** 1 Department of Medicinal Chemist and Pharmacognosy, College of Pharmacy, Qassim University, Qassim 51452, Saudi Arabia; m.omar@qu.edu.sa; 2 Department of Pharmacognosy, Faculty of Pharmacy, Al-Azhar University, Cairo 11371, Egypt; 3 Department of Pharmacology and Toxicology, College of Pharmacy, Qassim University, Qassim 51452, Saudi Arabia; m.azmi@qu.edu.sa (S.A.A.M.); su.mohammed@qu.edu.sa (M.S.M.S.); 4 Department of Biochemistry, Faculty of Medicine, Al-Azhar University, Assiut 71524, Egypt; 5 Department of Medicinal Chemistry and Pharmacognosy, Faculty of Pharmacy, JUST, Irbid 22110, Jordan; 6 Hospital of Police Academy, Nasr city, Cairo 11765, Egypt; mohamedshawky1974@yahoo.com; 7 Department of Pharmaceutical Analytical Chemistry, Faculty of Pharmacy, South Valley University, Qena 83523, Egypt; Adelpharma2004@svu.edu.eg; 8 Department of Pharmaceutics, College of Pharmacy, Qassim University, Qassim 51452, Saudi Arabia; A.Abdellatif@qu.edu.sa; 9 Department of Clinical Biochemistry, Faculty of Medicine, Umm Al-Qura University, Abdia, Makkah 21955, Saudi Arabia; syeid@uqu.edu.sa (S.Y.E.); mzreadi@uqu.edu.sa (M.Z.E.-R.); 10 Department of Biochemistry, Faculty of Pharmacy, Al-Azhar University, Assiut 71524, Egypt

**Keywords:** Sage, *Salvia officinalis*, cytotoxicity, hepatoprotection, MDA, TAOxC, MCF-7, HeLA cells, HepG-2 cells

## Abstract

Sage, *Salvia officinalis* L., is used worldwide as an aromatic herb for culinary purposes as well as a traditional medicinal agent for various ailments. Current investigations exhibited the effects of extended dryings of the herb on the yields, composition, oil quality, and hepatoprotective as well as anti-cancer biological activities of the hydrodistillation-obtained essential oils from the aerial parts of the plant. The essential oils’ yields, compositions, and biological activities levels of the fresh and differently timed and room-temperature dried herbs differed significantly. The lowest yields of the essential oil were obtained from the fresh herbs (FH, 631 mg, 0.16%), while the highest yield was obtained from the two-week dried herbs (2WDH, 1102 mg, 0.28%). A notable decrease in monoterpenes, with increment in the sesquiterpene constituents, was observed for the FH-based essential oil as compared to all the other batches of the essential oils obtained from the different-timed dried herbs. Additionally, characteristic chemotypic constituents of sage, i.e., α-pinene, camphene, β-pinene, myrcene, 1, 8-cineole, α-thujone, and camphor, were present in significantly higher proportions in all the dried herbs’ essential oils as compared to the FH-based essential oil. The in vivo hepatoprotective activity demonstrated significant reductions in the levels of AST, ALT, and ALP, as well as a significant increase in the total protein (*p* < 0.05) contents level, as compared to the acetaminophen (AAP) administered experimental group of rats. A significant reduction (*p* < 0.05) in the ALT level was demonstrated by the 4WDH-based essential oil in comparison to the FH-based essential oil. The levels of creatinine, cholesterol, and triglycerides were reduced (*p* < 0.05) in the pre-treated rats by the essential oil batches, with non-significant differences found among them as a result of the herbs dryings based oils. A notable increase in the viability of the cells, and total antioxidant capacity (TAOxC) levels, together with the reduction in malondialdehyde (MDA) levels were observed by the essential oils obtained from all the batches as compared with the AAP-treated cell-lines, HepG-2, HeLa, and MCF-7, that indicated the in vitro hepatoprotective effects of the sage essential oils. However, significant improvements in the in vivo and in vitro hepatoprotective activities with the 4WDH-based oil, as compared to all other essential oil-batches and silymarin standard demonstrated the beneficial effects of the drying protocol for the herb for its medicinal purposes.

## 1. Introduction

Sage, salvia, or *Salvia officinalis* L., the plant belonging to the family Lamiaceae, is a perennial aromatic herb cultivated worldwide for its medicinal, culinary, and flavor properties. The sage, as a culinary and medicinal herb, is popular worldwide and considered an economically important commodity for use in the apothecary and spices business. Herbal shops regularly trade sage as well as other aromatic herbs wherein the herbs are dried at room temperature under shade to retain their aromatic contents. The herb is worldwide available and is used in food preparations and also as an essential part of the tea mixture of the Mediterranean countries [1]. The folk-medicinal and traditional usage of sage is global in nature, and the Latin name of the plant “Salvia” means curative, which is indicative of the health-giving properties, and importance of this plant [2]. The aerial parts of the plant are used for the management of gout, rheumatism, diarrhea, and diabetes in the countries of Northern America, and Asia [3]. Sage is also used for the treatment of gastrointestinal disorders, such as dyspepsia and ulcers, as well as heartburn, and upper respiratory complaints, including sore throat, and inflammations [1]. Sage also has noticeable beneficial effects in age-related cognitive disorders [4]. It is strongly reputed to induce calmness and improve memory in Alzheimer’s patients [5]. The plant leaves are frequently used in the food industry due to their flavor and antioxidant, and antimicrobial properties [6]. The plant is also part of the perfumery and cosmetic industries [2].

Chemical analysis of the sage essential oil from different locations has been carried out, and detailed reports are available from different quarters. The major volatile constituents of the plant were determined as being α-thujone, 1,8-cineole, and camphor, which are considered the chemotypic constituents of the *S. officinalis* essential oil [7]. The effects of seasonal variations [8], geographical locations differences in oil quality and yields [8,9,10], essential oils’ extraction procedures [11,12,13], and the essential oils obtained through different drying methods [14,15] have been reported as parts of the parameters affecting the plant essential oils’ yields and constituents. Significant variations among sage essential oil constituents in response to the aforementioned parameters have been reported. The major chemotypic products of the sage essential oil, α-thujone, 1,8-cineole, and camphor also significantly varied in the essential oils obtained from the plant batches dried in an oven at 45 °C and 65 °C, and plant materials dried in a microwave oven (500 W) [14]. Likewise, the α-thujone and camphor’s componential ratios fluctuated in the essential oil batches obtained from the herbs collected in different seasons, and from different geographic locations [8].

Nonetheless, the essential oil compositions and yields obtained from *S. officinalis* have been extensively studied [7,16,17,18,19], and the effects of the extraction procedures, drying methods, and harvesting frequency on the plant’s essential oil constituents have been demonstrated in previous reports [14,15,20,21]. The current work instead, in contrast to the previous reports on the essential oils of sage from different locations, seasons, and multiple drying methods, dealt with the periodic effects of a single drying procedure that is frequently adopted by the apothecary and herbal dealers, i.e., room temperature and natural atmospheric pressure conditions for drying the herbs. To the best of information available, the effects of extended, i.e., 1- to 4-weeks, dryings on the sage essential oils yields and variations in their constituents have not been investigated. This is the first report disclosing the substantial effects of the plants’ drying effects on its oils’ yields, oils’ composition, and effects on the chemotypic constituents.

Hepatoprotective effects of herbal natural products have been reported, and several secondary metabolic origins compounds responsible for potential activity in treating liver dysfunctions are known [22]. The testing protocols, including animal models, in assessing the hepatoprotective activity are one of the important aspects in confirming the in vivo potential of the test material [23,24]. Moreover, for the cell lines–based assays for liver functional assessments, the HepG-2 cell lines are the prime choice, owing to their desirable biochemical and morphological characteristics imitating the normal hepatocytes; hence, they are used as a representative model for the in vitro hepatoprotective assays. In addition, the HepG-2 cell lines have a certain advantage over the normal hepatocytes in a way that they have high survival and maintenance rates in large quantities without changes in their drug-metabolizing enzyme activities, and this occurs in the primary cultures of the human hepatocytes [25]. Hence, the HepG-2 cell lines are widely used as an in vitro model for the assessment of various liver functions, their metabolic activity, and the evaluation of the drug’s toxicity [26]. In the context of hepatic disorders and hepatoprotective actions, acetaminophen (AAP, paracetamol) is widely used as an antipyretic, analgesic, and anti-inflammatory standard drug/agent. However, the AAP produces hepatotoxicity upon larger dose administrations, and the mechanisms responsible for in vivo liver toxicity of the AAP are complex [27], as it (AAP) undergoes metabolic activation in a cytochrome-P450–dependent step to produce a highly reactive metabolite, N-acetyl-*p*-benzoquinone imine (NAPQI), as well as free radicals, which can initiate lipid peroxidation. The in vivo toxicity induced by the AAP and the toxicity in cultured hepatocytes involves stimulations of lipid peroxidation, which is detected as an increase in the levels of malondialdehyde (MDA) formations [28].

The hepatoprotective effects of sage essential oil have been investigated by several research groups since the plant is claimed to be curative in nature with context to hepatoprotection, and is still used as part of the traditional medicament for cure and protection of liver-related disorders in several quarters [29,30,31,32]. Previous reports describing the cytotoxic activities of sage essential oils are also available [33,34,35].

The current study specifics were performed to evaluate the impacts of extended dryings of the sage aerial parts on their essential oil yields and constituents’ variations, as well as effects on biological activities, in comparison to the essential oil obtained from the fresh herb. Specifically, the current study investigated the variations in the yields and chemical compositions of the essential oil batches, and their impact on cytotoxicity in MCF-7, and HeLa cell lines, together with the hepatoprotective activity against AAP-induced liver damages in the in vivo models in Wistar male rats, and in an in vitro model in HepG-2 cell lines.

## 2. Materials and Methods

### 2.1. Plant Materials

The aerial parts of the sage herb were collected in February 2019 from the gardens maintained by the Ministry of Agriculture, Qassim province, KSA. The institutional botanist identified the plants as *Salvia officinalis* L. The fresh plants’ aerial parts were divided into 5 equal groups of 400 g each. One group of the fresh aerial parts of the plants were subjected to hydrodistillation, while other groups were dried in shade at room temperature (25 ± 2 °C) for a pre-defined time (one-week intervals) as defined in Section 2.2.

### 2.2. Dryings of the Plants and Hydrodistillation for Obtaining Essential Oil Batches

The five fresh herb groups of 400 g each were individually subjected to the procedure as follows. Batch one (fresh aerial parts herb group, 400 g) of the plant was mixed with 700 mL of distilled water in a 2 L round-bottom flask, and instantly subjected to hydrodistillation for 5 h using a Clevenger apparatus. The subsequent batches, two, three, four, and five (400 g each) were allowed to dry at room temperature (25 ± 2 °C) in shade for one, two, three, and four weeks, respectively, before their hydrodistillation to procure the essential oils in the same conditions as used for the fresh plants batch. The obtained essential oils were dried over anhydrous sodium sulfate, and the percent yields of the essential oils for each batch was calculated according to the following formula:

(1) $$Oil\;Yields\;\%=\left(\frac{A}{400}\right)\times 100$$

where *A* is the amount of essential oil in gram obtained from 400 g of the fresh aerial parts of the plant. The batches’ weight reductions were calculated after the defined drying periods for each batch.

### 2.3. Gas Chromatography-Flame Ionization Detector (GC-FID) Analyses

Hydrodistilled essential oil batches of the herb sage were subjected to GC-FID analyses on a Perkin Elmer Auto System XL gas chromatography instrument equipped with a flame ionization detector (FID). The chromatographic separations of the essential oil samples were achieved on a fused silica capillary column ZB5 (60 m × 0.32 mm i.d. × 0.25 µm film thickness). The oven temperature was maintained initially at 50 °C and programmed from 50 °C to 240 °C at a rate of 3 °C/min. Helium gas was used as a carrier at a flow rate of 1.1 mL/min. The injector and detector temperatures were 220 °C and 250 °C, respectively.

### 2.4. Identification of the Essential oil Constituents

The essential oils obtained from the fresh sage and the dried batches were identified based on the experimental retention index (*RI*) calculated with references to standard *n*-alkenes series (C_8_–C_40_), and the retention indices reported in the literature under similar GC experimental conditions. Additionally, the identification of the compounds was carried out based on their retention time and comparisons with the mass spectral libraries (National Institute of Standards and Technology (NIST-11) and Wiley Database). The relative percentages of the constituents were calculated from the area under the peak obtained from the GC-FID chromatogram.

### 2.5. In Vivo Hepatoprotective Assay

#### 2.5.1. Experimental Animals

The in vivo hepatoprotective activity of the sage essential oil batches was performed using Wistar male rats weighing about 200–250 g, which were kindly provided by the animal house facility of the College of Pharmacy, Qassim University. The rats were housed in suitable humidity and temperature (25 ± 2 °C) and given a standard diet and water *ad libitum*. The animals were kept in a pathogen-controlled, air-conditioned room in the animal house. All the experiments were performed, according to the guidelines for animal studies that were approved by the Ethical Committee of College of Pharmacy, Qassim University, KSA.

#### 2.5.2. Acute Toxicity Studies

Briefly, ten-weeks-old male Wistar rats (*n* = 15), weighing 200–250 g, being overnight fasted, were weighed, and a single dose of 50, 100, and 200 mg/kg (*n* = 5/group) of *Salvia officinalis* essential oil was administered, using the oral route. The animals were observed for abnormality in behavior and movements for the first three days and mortality for up to two weeks. Based on the results, 20 mg/kg, 10% of the maximum administered dose according to the Hedge and Sterner scale, was selected for evaluation of the hepatoprotective activity [36].

#### 2.5.3. Animal Groups

A total of 40 ten-week-old Wistar male rats, weighing 200–250 g, were divided randomly into eight equal groups (*n* = 5); the first group was considered the control and received oral supplementation of saline, using an orogastric cannula. The second group of animals (negative control) received oral administration of AAP (in a dose of 500 mg/kg) once daily starting from the 11th day of the experiment for 5 consecutive days to induce liver injury. The third, fourth, fifth, sixth, and seventh groups of animals received 20 mg/kg BW (bodyweight), once daily, for 15 days the essential oils obtained from the fresh herb (FH), one-week (1WDH), two-week (2WDH), three-week (3WDH), and four-week dried herb (4WDH) of the *Salvia officinalis,* respectively. The animal groups (third to seventh groups) received AAP to induce liver injury (in a dose of 500 mg/kg) starting from the 11th day of the experiment for 5 days. Standard liver support was administrated to group number eight, which was pretreated with silymarin (oral dose: 100 mg/kg, 15 days), and AAP for the last 5 days. At the end of the experiment, blood samples were collected by retro-orbital puncture, serum was separated from all groups’ collected blood for the determination of liver functions (ALP, AST, ALT, and total protein) as well as kidneys functions (urea and creatinine) and the lipid profile (triglycerides and total cholesterol) analyses.

#### 2.5.4. Determination of Liver, Kidneys Functions, and Lipid Profile

The plasma levels of ALT, AST, ALP, and creatinine were determined, using an optimized UV-test, according to the international federation of clinical chemistry (IFCC). The plasma levels of total protein, urea, cholesterol, and triglycerides were measured, using the colorimetric methods. All the reagents were provided by the Crescent Diagnostics Company, KSA.

### 2.6. Cell-proliferation Assays

Human breast adenocarcinoma cell lines (MCF-7), cervical carcinoma cell lines (HeLa), and hepatocellular carcinoma cell lines (HepG-2) were used to evaluate the cytotoxic activity of the different batches of the sage essential oils in comparison with the normal fibroblast lung cell lines, MRC-5. The cell lines were grown in RPMI1640 or DMEM media (Gibco) supplemental with 10% fetal bovine serum (FBS) (Gibco), penicillin-streptomycin 1%, and L-glutamine 1%. Cells were grown under optimum growth conditions at 37 °C in a humidified atmosphere of 5% CO_2_. The proliferation of tested cells after treatment with the sage essential oils from different batches and doxorubicin as a positive cytotoxic drug was determined by the colorimetric 3-(4,5- dimethylthiazol-2-yl)-2,5-diphenyl tetrazolium bromide (MTT) assays. Cells (1 × 10^4^ cells/well) were incubated in 96-wells plates for 24 h. After that, the cells were further incubated for 24 h with and without essential oils and doxorubicin. MTT solution (Sigma-Aldrich, Germany) (10 μL of 5 mg/mL in PBS) was added to each well and incubated for a further 4 h at 37 °C in a CO_2_ incubator. The formazan crystals were dissolved in 100 μL of DMSO, and the color density was measured at 570 nm using SpectraMax M II microplate reader (Molecular Devices, LLC. San Jose, CA, USA).

#### Viability percentage and Selectivity Index:

To calculate the viability percentage, the following formula was used:

Viability % = (OD _treatment_ /OD _control_) × 100 (2)

IC_50_ values (i.e., the sample concentration that exerts 50% inhibition concerning untreated cells) were determined for all the cell lines. The selectivity index (SI), which indicates the cytotoxic selectivity (i.e., drug safety) for tested samples against cancer cells versus normal cells, was calculated from the following formula:

SI = IC_50_ calculated for normal cells/ IC_50_ calculated for cancer cells (3)

The SI values higher than 2 were considered to be high selectivity [37].

### 2.7. Acetaminophen (AAP) Induced Hepatotoxicity in Hepg2 Cells

To evaluate the hepatoprotective effects of the sage essential oils on HepG-2 cell lines, the non-toxic concentration of the sage essential oil, 100 μg/mL, was selected to conduct the hepatoprotective activity assessment using the MTT assay as described earlier (Section 2.6). The HepG-2 cell lines were treated with AAP (4 mM) for 24 h, and the HepG-2 cells in the medium were considered as a negative control. Further, the hepatoprotective assays were performed using standard methods as described in each assay in the following sections as detailed below. The HepG-2 cells were pretreated with the essential oils for 12 h and incubated with AAP (4 mM) for 24 h, with and without the essential oils; the HepG-2 cells in medium only were considered as a negative control.

### 2.8. Measurement of Total Antioxidant Capacity (TAOxC)

The HepG-2 cell lines were treated with AAP in the presence and absence of the sage essential oils obtained from different batches. After treatment, the cells were lysed and suspended by sonication on ice in 0.9% sodium chloride solution containing 0.1% glucose and 5 mM potassium phosphate buffer (pH 7.4). The supernatant of the lysed cells was used to measure TAOxC, using an antioxidant assay kit obtained from Cayman Chemical Company (Ann Arbor, MI, USA). The assay was dependent on the ability of the antioxidants in the sample to inhibit the oxidation of 2,2’-azino-bis-3-ethylbenzothiazoline (ABTS) to ABTS^+^ by metmyoglobin absorbance in the wells, which were measured after 5 min at a wavelength of 405 nm on a microplate reader, SpectraMax M II (Molecular Devices, LLC. San Jose, CA, USA). The results were expressed as millimoles of the antioxidants utilized [38].

### 2.9. Measurement of MDA for Lipid Peroxidation

Malondialdehyde (MDA), an end product of the lipid peroxidation, was used as an oxidative stress marker, and its concentration was measured using a thiobarbituric acid reactive substance (TBARS) assay kit obtained from the Cayman Chemical Company. The HepG-2 cells were treated with AAP in the presence and absence of sage essential oils, the supernatant of cells lysate or the standard sodium dodecyl sulfate, and the color reagent was added, heated to 100 °C for 1 h, and immediately cooled in an ice bath and centrifuged. The absorbance of the product was measured at a wavelength of 540 nm on a microplate reader, SpectraMax M II (Molecular Devices, LLC. San Jose, CA, USA). The extent of lipid peroxidation was quantified by estimating the MDA concentration. The results are expressed as micromoles of MDA equivalents formed per liter.

### 2.10. Statistical Analysis

The results were analyzed using GraphPad Prism V6 (GraphPad Software, San Diego, CA, USA). Data were expressed as mean ± SD. of 3–4 independent experiments performed at least in triplicate. One-way analysis of variance (ANOVA) followed by Tukey’s test was used to detect any significant differences among the different mean values. A *p*-value less than 0.05 was considered a significant difference.

## 3. Results and Discussion

### 3.1. Sage Essential Oil Obtained from the Fresh Aerial Parts of the Plants and the Extended-Dried Plant Batches

The current study was designed to evaluate the effects of extended dryings on the sage essential oil yields, compositions, and biological activities, wherein the herbs’ aerial parts were utilized to obtain the essential oils by the hydrodistillation process. The factors of drying temperatures (25 ± 2 °C), pressure (atmospheric pressure), and the amount of the fresh herbs (400 g) in each batch were constants; however, the variable parameter was the drying period and the weight loss of the dried herbs. From the viewpoint of essential oils production, the overall results in Table 1 show higher essential oil yields through the hydrodistillation method from the dried aerial parts of the herbs batches than that obtained from the fresh herb.

The results showed a noticeable change in the plant weight after one week of drying from 400 g to 131 g (−67.25%) and a significant increase in the essential oil yields obtained from the one-week dried herb (923 ± 6.34 mg) as compared to the fresh herb material (631 ± 8.05 mg). The herbs dried for two weeks showed a drastic change in their weight; a loss of the weight from 400 g to 131 g, as compared to the fresh sample, was observed. At the same time, the essential oil yields percentage was ~75% higher for the two weeks dried herbs batch than the essential oil yields obtained from the fresh herbs sample. The results in Table 1 displayed that further dryings of the sage for three and four weeks were accompanied by a reduction in the yields of the essential oils in comparison to the two weeks of dried herbs, which showed maximum yields of the essential oil. The weight reduction in response to the extended dryings was a logical culmination of the drying process; nonetheless, the improvements in the essential oil obtained from the herbs after two weeks of drying as compared to the fresh plant-based oil yields need careful consideration. The reduction in the water to essential oil ratio in the herbs during the drying process is a plausible explanation for the higher yields of essential oil recovery from the dried herbs rather than the fresh herbs batch. The drying period extension over two weeks might be accompanied by higher emission of the essential oil from the herbal materials, which possibly explains the significant reduction in the essential oil yields obtained from the dried herbs batches of three and four weeks as compared to the two weeks dried herbs. Notably, similar results in parallel to the current findings for weight loss and essential oil yields were recently reported for rosemary, *Rosmarinus officinalis* [39]. Nevertheless, the essential oil yields percentages from the fresh and extended dried sage materials ranged from 1.5 to 2.8 mL/Kg, and it did not confirm the recommendations of European Pharmacopoeial (EP) standards for *Salvia officinalis* oil yields (10 mL/Kg) [19]. However, the essential oil yields were in alignment with the reported values for the essential oil yields from the aerial parts of the plant, sage, grown in the Middle Eastern regions, which varied between 0.8% to 2.5% [9,40,41,42]. Additionally, the combined results of the Table 1 confirmed the economic importance of the herbs-drying process for better essential oils production. The results also are in agreement with the normal practice conducted by the herbalist, and home drying of the herbs utilized for different culinary purposes.

### 3.2. Componential Analysis of the Essential Oil Obtained from Different Batches

The essential oils obtained from all the batches of sage (fresh and dried aerial parts) were analyzed for the presence, in percentages, of their constituents (GC-FID analysis, supplementary file, Table S1–S3). The results exhibited in Table 2 demonstrated distinct differences between the constituents and their percentages in all five batches of sage oil. Among all the identified constituents, five components were identified only in the fresh herbs–based essential oil, i.e., butyl acetate (0.64%), α-phellandrene (0.3%), neral (0.17%), α-cadinene (0.17%), and viridiflorol (0.59%). The differently timed dried herbs exhibited an absence as well as an increment and/or reduction in percent ratios of different components in the essential oils (Table 2). The results showed major differences in the percentages of the chemotypic constituents of the essential oils obtained from the dried herbs–based essential oils as compared to the fresh herb-based essential oil. The non-oxygenated monoterpenes, i.e., α-pinene, camphene, β-pinene, myrcene, and γ-terpinene, were significantly increased (*p* < 0.05) in the dried herbs–based essential oils batches, as compared to the essential oil obtained from the fresh herb. Here, the α-pinene concentration was significantly increased (*p* < 0.05) from 0.07% of the fresh herbs–based essential oil to 1.54, 1.57, 1.46, and 0.73% in the one-, two-, three-, and four weeks dried herbs–based essential oils. Some of the oxygenated monoterpenes, e.g., 1,8-cineole, α-thujone, and camphor, were also significantly increased (*p* < 0.05) in the essential oils batches obtained from dried sage as compared to the essential oil batch obtained from the fresh herbs (Table 2). In contrast, the sesquiterpenes β-caryophyllene, α-humulene, d-cadinene, humulene epoxide III, 14-hydroxy-(Z)-caryophyllene, and α-bisabolol showed drastic reductions (*p* < 0.05) in their contents ratio in the oil yields of the dried herbs–based essential oil batches in comparison to the fresh herbs–based essential oil. For instance, the α-bisabolol percentage was reduced from 3.43% in the fresh herbs–based essential oil to 0.27% in the essential oil batch of the one-week dried herbs, which equals to a 92.12% reduction in the percentage of the α-bisabolol. However, *p*-cymene-8-ol and dihydrocarveol acetate monoterpenes were found in higher concentrations in the fresh herbs–based essential oil.

**Figure 1:**
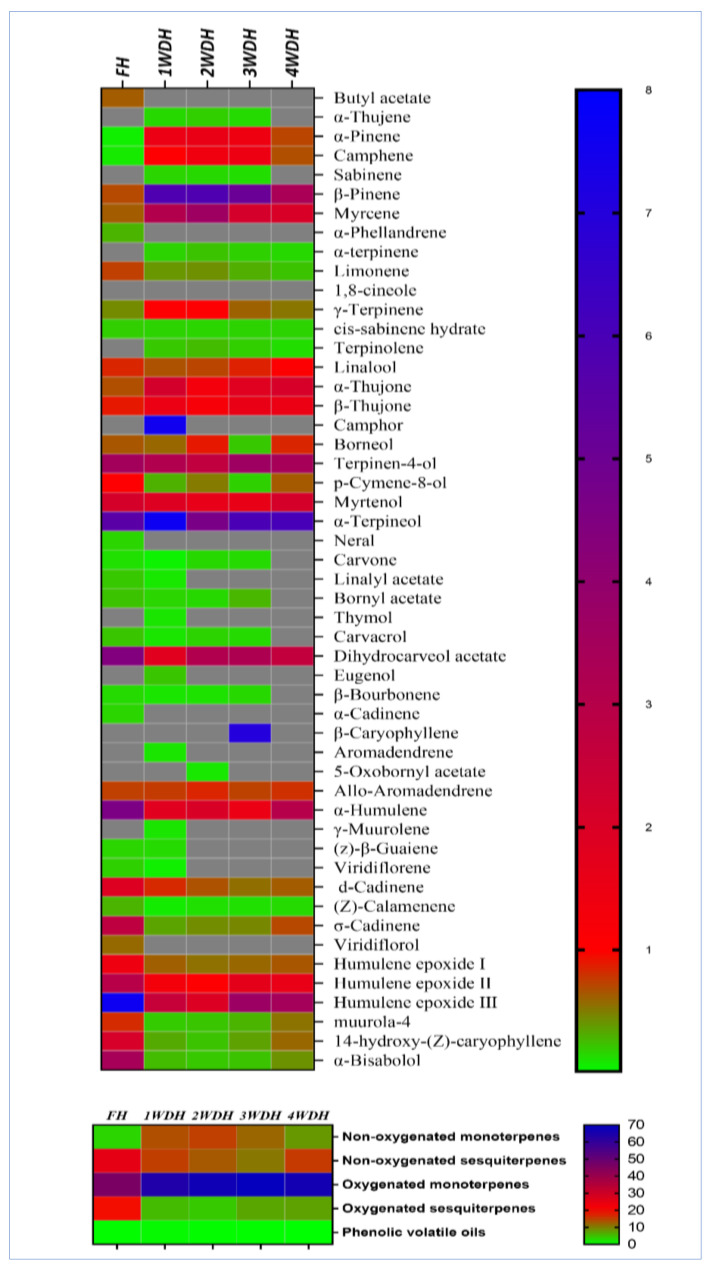
A heat-map comparison based on the abundance of an individual component of sage’s essential oils from different batches; gray color indicates un-detected from the identified component.

The overall follow-up of the decreasing and increasing percentages of the sage essential oils’ constituents among the fresh and dried herbs reflected that most of the monoterpene constituents, including the oxygenated and non-oxygenated, were steadily increasing for up to three weeks of drying as compared to the fresh herbs–based essential oil (Table 2, Figure 1). Interestingly, the percentages of the sesquiterpenes were also decreased in the dried herbs batches of the sage essential oils, as compared to the fresh herbs–based essential oil batch. Wherein, the fresh herbs–based essential oil analysis showed 24.82% and 18.96% as compared to 15.03% and 5.27% of the non-oxygenated and oxygenated sesquiterpenes, respectively, which was obtained after only one week of drying as shown in the heat-map comparison which is based on the abundance of major components found in the essential oil batches (Figure 1). The number of identified compounds in every essential oil batch was also one of the distinct comparable points among them. The highest numbers of constituents in the essential oil obtained from the fresh, and one-week dried herbs based essential oil batches of the plant (46 compounds representing 98.3%, and 98.43% of the essential oil, respectively), as compared to the 40 and 39 identified constituents in the two- and three-weeks dried herbs–based essential oil batches, which were represented at 99.37% and 99.26% of the essential oil weight ratios according to the peak area analyses, respectively. Nonetheless, the drastic reductions in the identified compounds were shown in the four-week dried herbs–based essential oil, which showed 33 constituents and represented 97.14% of the essential oil componential weight. Moreover, all the common sage constituents were identified in the fresh herbs-based essential oil batch and the batches obtained after dryings of the herbs. A number of 46 constituents were identified in the fresh herbs and one week dried herbs based essential oils, while the two and three weeks dried herbs based oil contained 40 and 39 constituents, respectively, which formed their total yields of the oils, and their identified and the unidentified constituents weight ratios. The four weeks dried herb-based essential oil contained only 33 constituents in it (Table 2). The GC-FID analysis also revealed that the chemotypic constituents of the sage essential oils were represented in all the essential oil batches as major constituents with different proportions. For instance, 1,8-cineole was found as a major component; however, its proportions were significantly different, and vacillating in different essential oil batches. The economical outcome that could be implied from the results demonstrated in Table 1 and Table 2, for the essential oil yields percentage and componential representation of the essential oil constituents in each essential oil batch, showed the importance of sage drying for two weeks in shade at room temperature before the procurement of its essential oils. However, the biological activities of these essential oil batches were another factor mostly centered on the biological activity of the constituents rather than the amount of the obtained essential oil yields from each batch.

### 3.3. Hepatoprotective Effect of the Essential Oil Batches

The quality of sage essential oils obtained from fresh and different-timed dried batches, i.e., 1WDH, 2WDH, 3WDH, and 4WDH, of the herbs were evaluated for their effectiveness to restore the liver and kidneys functions, together with the lipid profile in comparison to the normal behavior, which was impaired by AAP-induced liver toxicity in the experimental animal models, rats. The ability of sage’s essential oils from different batches to protect the HepG-2 cells toxicity induced by AAP was also evaluated as an in vitro hepatoprotective assay. The sage decoction was reported to exaggerate the liver toxicity by the CCl_4_ in the mice models [43], which is an unexpected result of the sage infusion. However, the hepatoprotective activity of sage infusion against the liver injury inducer, azathioprine, was reported in rats [44]. Sage’s essential oil and herbal extract were also reported to protect against liver injury induced by AAP, attributed to the antioxidant potentials of the sage constituents [29,45].

#### 3.3.1. In vivo Hepatoprotective Effect

The current study showed a significant increase in the serum levels of AST, ALT, and ALP in a concomitant manner with a significant decrease in the total protein contents in the AAP-induced liver injury in several groups of animals, as compared to the control group. These disorders occurred as a result of AAP-induced liver toxicity, which led to hepatic cell damage and necrosis [46] culminating from reactive oxygen species accumulation, lipid peroxidation [47], and calcium release [48], which represented a plausible biomechanism of the AAP-induced liver injury. The pre-treated rats by sage herbs’ essential oils showed a significant decrease in AST, ALT, ALP, and a significant increase in total protein contents (*p* < 0.001) in comparison to the AAP-induced liver injury in groups of animals under study. Similar results were obtained from the silymarin-treated rats, which is considered a standard liver support drug which restores hepatic cells functions in liver injury conditions induced by the drug toxicity, and the interplay of free radicals [49]. These findings are in complete alignment with previous reports [29,50], wherein the sage essential oil showed hepatoprotective effects, due to its antioxidant potential. Recently, another study reported that sage essential oil reduces oxidative stress and the toxic effects in liver injury which was induced by vanadium metal in rat liver [31]. Similarly, another study observed that sage protected the liver against isoniazid-induced hepatic toxicity [51]. In this regard, the *Salvia officinalis* leaves’ methanolic extract also protected the liver injury induced by aflatoxins [52]. The possible hepatoprotective mechanism of sage may be attributed to its high contents of the oxygenated mono- and sesquiterpenes, e.g., 1,8-cineole, camphor, and humulene epoxide II, that were detected in high concentrations in the current study. These compounds are reported to have antioxidant effects as well as free radical scavenging properties and are in abundance in higher quantities in the hepatoprotective essential oils from other plants also [53,54]. In contrast, a previous study also reported that *Salvia officinalis* aggregated the CCl_4_-induced liver toxicity in mice, and the bio-mechanism for it can possibly be attributed to herbs–drug interactions [43]. There were no significant differences detected in the liver functions of all the essential oils (1WDH–4WDH batches) administered animal groups, except that the 4WDH-dried herb-based essential oil showed a significant decrease in the ALT enzymatic activity in comparison to the fresh herb-based essential oil (*p* < 0.05). These effects may be attributed to the presence of 1,8-cineole, and camphor, accumulated as a result of the herbs drying. Further investigations are needed to clarify the issue (Table 3).

The present work showed significant increase in the serum levels of cholesterol, triglycerides, urea, and creatinine on the AAP-administered groups, as compared to the control group (*p* = 0.001***; *p* < 0.001***; *p* < 0.01*** and *p* < 0.001***), respectively. The observations are in tune with the outcomes of AAP-induced toxicity also documented previously [46,47,48]. The current study showed a significant decrease in the cholesterol, triglycerides, and creatinine levels in the sage’s essential oil pre-treated and silymarin-treated groups, with a *p*-value (0.001 ***), as compared to the AAP-administered groups. Additionally, no significant differences were detected in the kidneys’ functions, cholesterol, and triglycerides levels among all the animal groups when compared for the use of oil obtained from herbs drying (1WDH–4WDH). These observations are commensurate with the study which reported that *Salvia officinalis* use normalizes the lipid disturbance, and lipoprotein metabolism in rat liver injury induced by vanadium metal [31]. Sage also decreased cholesterol, triglycerides, and creatinine levels in streptozotocin-induced diabetic rats [55] (Table 4).

#### 3.3.2. In vitro Hepatoprotective Effects

**Figure 2:**
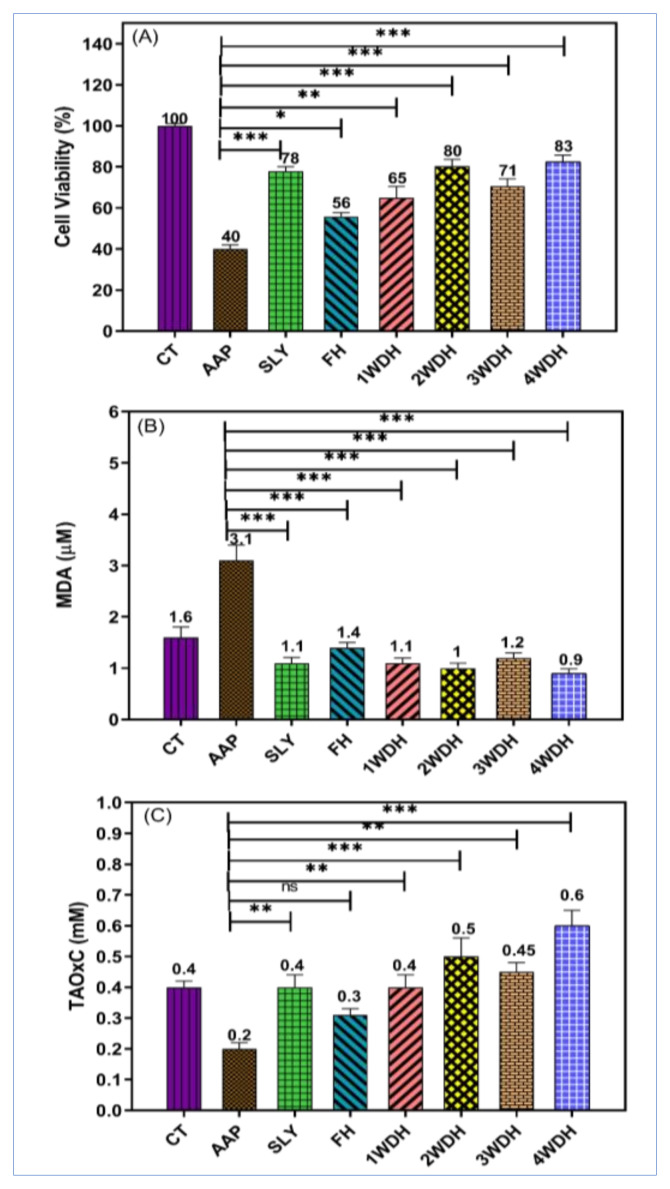
Hepatoprotective effects of sage essential oils against damage induced by 4 mM acetaminophen (AAP) in HepG-2 cells for 24 h in comparison to silymarin. The cytotoxicity of AAP with and without selected dose (100 μg/mL < IC_50_ values) of sage essential oils and silymarin (SLY) on hepatic cell lines (HepG-2) (**A**) for hepatoprotective activity tests MDA levels (μM) (**B**), and TAOxC levels (mM) (**C**) in HepG-2 cells after exposure to 4 mM AAP and pretreated with sage essential oils or silymarin. Controls: supplemented media (CT); AAP 4 mM (AAP), silymarin (100 μg/mL) (SLY). Values are the mean  ± SD of three independent experiments performed in triplicate. * For *p* < 0.05, ** for *p* < 0.01, and *** for *p* < 0.001.

The current study used an in vitro cell culture model (HepG-2 cells) to evaluate the hepatoprotective activity of the fresh and differently timed dried sage herbs–based essential oils obtained by the hydrodistillation procedure against liver damages induced by the AAP. The strategy was used to evaluate the hepatoprotective effects of the sage’s essential oil and to support the findings obtained from in vivo studies. The cytotoxic effects of AAP were determined in the presence, and absence of the essential oils obtained from the fresh and other differently timed dried herbs–based essential oils as well as with the standard hepatic support, silymarin (Figure 2A).

The cytotoxic activity results of the current study demonstrated that the selected doses of sage essential oils were non-toxic at 100 μg/mL concentrations. It was also found that the sage’s essential oil significantly improved the viability of the cells of AAP-treated HepG-2 from 40% to 56% by FH, to 65% by 1WDH, to 80% by 2WDH, to 71% by 3WDH, and 83% by 4WDH as compared to the 78% viability of the silymarin-treated animals group (Figure 2A).

The hepatoprotective effects of the sage essential oils on HepG-2 cells that were pretreated with a hepatoprotective agent, and subsequently exposed to APP to induce damage are shown in Figure 2. The pretreated HepG-2 cells with FH, 1WDH, 2WDH, 3WDH, and 4WDH essential oils significantly decreased the MDA levels of the AAP treated cells from 3.1 μM to 1.1, 1.4, 1.1, 1 and 1.2 μM, respectively. In addition, a significant increase in the TAOxC levels of the AAP-treated cells from 0.2 mM to 0.4, 0.3, 0.5, 0.45, and 0.6 mM, respectively, was observed. Furthermore, the pretreatment with silymarin significantly decreased the MDA levels to 1.1 μM as well as an increase in TAOxC levels to 0.4 mM of the AAP-treated HepG-2 cells.

The exposure of HepG-2 cells to AAP demonstrated a significant reduction in the viability of the cells as indicated by their inability to metabolize the tetrazolium salt. A significant decrease in TAOxC, as well as a significant increase in the levels of MDA (Figure 2B,C), was detected. The underlying mechanisms of the in vitro liver damage caused by the AAP may be attributed to the AAP concentration and the exposure time [38]. The HepG-2 cells were exposed to the toxic dose of AAP that led to the generation of reactive oxygen species (ROS) interacting with the macromolecules inside of the cells [56]. This interaction results in DNA damage, lipid peroxidation of the lipids bilayers of the cell membrane, as well as denaturation of many essential proteins of the cells, and finally, exhibits cells death as observed in the loss of 40% of the viability of the cells by treatment with 4 mM of AAP. The exposure of hepatic cell lines to a high concentration of AAP causes cells injury and reduces viability as also reported previously [57]. The balancing between the oxidant and antioxidant capacities inside of the cells is important for the cells’ survival. Therefore, two parameters, MDA and TAOxC, including the cell viability, were evaluated to assess the hepatoprotective effects of all the essential oils batches obtained from sage. MDA is a biomarker of ROS effects, especially lipo-peroxidation, and TAOxC is an indicator marker for the general antioxidant status of cells.

Oxidative stress plays a major role in AAP-induced toxicity as observed by decreases in the TAOxC, and an increase in the MDA levels after treatment of HepG-2 cells with AAP. Several studies have suggested that the oxidative stress that leads to apoptosis is the cause of cell death in the HepG-2 cell lines. It was found that the pre-treated HepG-2 cells with different essential oils (100 μg/mL) obtained in the current study showed significant improvements in the cell viability. It also showed an increase in the TAOxC and a reduction in the MDA levels (Figure 1). These results suggest that the sage essential oil exerts hepatoprotective effects in AAP-induced damages in the HepG-2 cell lines. It is presumed that the hepatoprotective effects of the sage essential oil are mainly owing to their antioxidant contents, i.e., 1, 8-cineole, β-pinene, camphor, β-caryophyllene, and α-pinene. The significant improvements in the HepG-2 protective effects demonstrated by the essential oils obtained from differently-timed dried herbs, especially the 4WDH, as compared to the FH-based essential oil of the sage herbs. This can be attributed to the significant increase in the 1,8-cineole, β-pinene, camphor, and α-pinene presence in the dried essential oil batches as compared to the FH-based essential oil. Notably, the results also confirmed the in vivo observations, wherein the 4WDH-based sage essential oil significantly decreased the ALT enzymatic activity compared to the essential oil obtained by the FH (*p* < 0.05). It was also revealed that the 4WDH-based essential oil-induced significant elevation of TAOxC as compared to the standard hepatoprotective drug, silymarin. These effects seemed attributed to the cumulative effects of the major essential oil constituents in the 2WDH- and 4WDH-based essential oils that possessed comparatively strong antioxidant activity, owing to the higher contents of the constituents, e.g., 1, 8-cineole, and camphor. All the dried herb-based essential oil batches significantly increased the TAOxC. However, the 1WDH and 3WDH essential oils showed comparable results to the silymarin-treated cells. Similar results were also obtained for the levels of MDA, which were significantly reduced in the cells treated by the silymarin and the dried herbs–based essential oil batches, compared to the fresh sage essential oil. The fresh sage essential oil also showed a significant reduction in the MDA levels as compared to the AAP-treated cells.

### 3.4. Anticancer Effects of Essential Oils Obtained from Different-Timed Drying Herbs Batches

**Figure 3:**
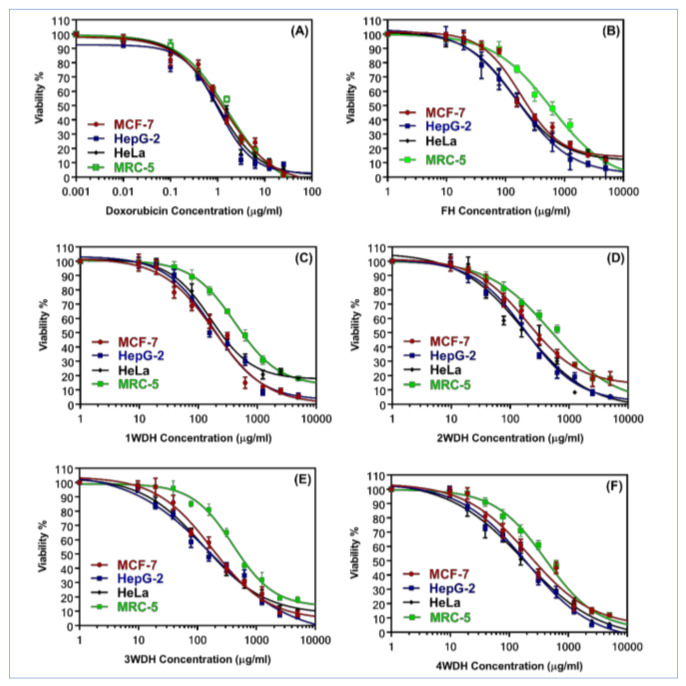
Dose-response curve of cytotoxicity of doxorubicin as a positive control (**A**), sage essential oils (1–500 μg/mL) of FH (**B**), 1WDH (**C**), 2WDH (**D**), 3WDH (**E**), and 4WDH (**F**) on cancer cell lines breast (MCF-7), hepatic (HepG-2), and cervical (HeLa) in comparison to normal cells (MRC-5).

The effects of the sage essential oil obtained from the fresh herbs, and dried herbs were evaluated by the MTT assay for the cell viability of cancer and normal cell lines. The results showed that all the essential oil batches from sage showed moderate cytotoxicity against cancer cell lines; hepatocellular carcinoma HepG-2, cervical carcinoma HeLa, and the breast carcinoma cell lines, MCF-7 (Table 5). Figure 3 represents the dose-response curve of the sage essential oil batches on the viability of all the cell lines used in this study. As shown in the curves, sage’s essential oils inhibited all the cancer cells, HepG-2, HeLa, and MCF-7, in a dose-dependent manner (1–500 μg/mL), while the curves showing an upgrade pattern indicated the low cytotoxicity of the tested samples on the normal cells (MRC-5).

**Figure 4:**
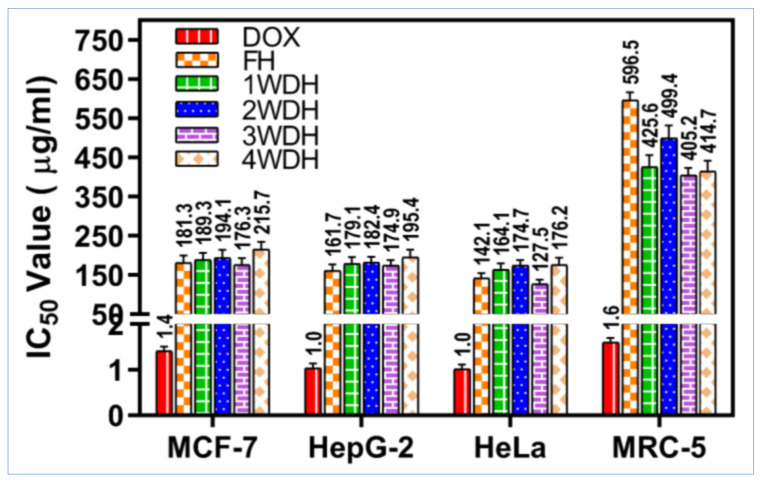
The cytotoxicity IC_50_ values of the sage essential oils (μg/mL) on cancer cell lines breast (MCF-7), hepatic (HepG-2), and cervical (HeLa) in comparison to the normal cells (MRC-5). Doxorubicin was used as a cytotoxic drug (positive control).

Figure 4 shows the summarized comparisons between the IC_50_ values of the tested samples and the positive control, doxorubicin, on the cancer cell lines, and the normal fibroblast cells. The 4WDH essential oil showed the lowest cytotoxicity on all the tested cancer cell lines, i.e., HepG-2, HeLa, and MCF-7, with IC_50_ values ranging from 176.2 μg/mL for HeLa cells to 215.7 μg/mL for the MCF-7 cell lines. The FH-based essential oil seemed to be having the highest cytotoxic effects on the cancer cells, and the lowest cytotoxic effects on the normal cells with the highest SI: HeLa (IC_50_ = 142.1 μg/mL, SI = 4.2), HepG-2 (IC_50_ = 161.7 μg/mL, SI = 3.7), and MCF-7 cells (IC_50_ = 181.3, SI = 3.3) (Table 5). A previous study also demonstrated the ability of the *S. officinalis* polar solvents extracted material to inhibit MCF-7 cancer cell lines without significant cytotoxic effects against normal human umbilical vein endothelial cells with SI > 3, and this is in agreement with our current findings [58].

## 4. Conclusions

This report comprehensively demonstrated the extended drying effect of aerial parts of *S. officinalis* on oil yields, its quality, and its effects on biological activities. Sage yielded the highest yields of essential oil from the two-week shade and room-temperature dried plant materials, with 0.28% of essential oil procurement containing over 99.82 ± 0.18% of the sage oil’s reported componential constituents. The typical chemotypic components were present in abundance, and presumably are part of the active constituents of the essential oil against induced liver damages and active cancer cell lines, i.e., MCF-7, HepG-2, and HeLA. The herb and its essential oil exhibited beneficial properties and are recommended for use in liver disorders. Their prophylactic efficacy in the liver damage caused by acetaminophen lends credence to their widespread use in culinary purposes worldwide. The essential oil yields modulation study from the differently timed drying of fresh herbs established the preferable drying period of two weeks for maximum essential oil yields from the herbs. Nonetheless, the essential oil obtained from two-week dried sage herbs also exhibited better liver protection, as well as anti-cancer activities in the in vitro conditions. The potential for bulk scale essential oil procurement from the herbs and its household use for perceived health benefits were thus verified.

## Figures and Tables

**Table 1:** Reduction in sage herbs’ weights and essential oils obtained by hydrodistillation in response to extended dryings.

Periods of Drying	Fresh Weight	Weight after Drying	Essential Oil (mg)	% Yields *
Fresh Herb (FH)	400 g	400 g	631 ± 8.05	0.16
1WDH		131 g	923 ± 6.34	0.23
2WDH		111 g	1102 ± 15.58	0.28
3WDH		107 g	944 ± 5.73	0.24
4WDH		107 g	702 ± 9.10	0.18

* Yield percentages were calculated from the equation: weight of the essential oil obtained in gram/ 400 × 100.

**Table 2:** Chemical constituents of the *Salvia officinalis* essential oils batches distilled from the fresh aerial parts of the herbs and the aerial parts after different drying periods.

No.	Components	KI^exp.^	KI^rep.^	FH	1WDH	2WDH	3WDH	4WDH
1	Butyl acetate	815	817	0.64 ± 0.09				
2	α-Thujene	930	932		0.16 ± 0.02	0.20 ± 0.01	0.15 ± 0.01	
3	α-Pinene	938	939	0.07 ± 0.02 ^A^	1.54 ± 0.14 ^B^	1.57 ± 0.06 ^B^	1.46 ± 0.05 ^B^	0.73 ± 0.08 ^B^
4	Camphene	954	950	0.09 ± 0.02 ^A^	1.01 ± 0.05 ^B^	1.40 ± 0.08 ^B^	1.49 ± 0.08 ^B^	0.69 ± 0.05 ^B^
5	Sabinene	977	976		0.17 ± 0.02	0.16 ± 0.01	0.14 ± 0.01	
6	β-Pinene	983	980	0.71 ± 0.08 ^A^	5.81 ± 0.56 ^B^	5.82 ± 0.24 ^B^	5.08 ± 0.22 ^B^	3.33 ± 0.13 ^B^
7	Myrcene	992	992	0.64 ± 0.08 ^A^	3.10 ± 0.23 ^B^	3.64 ± 0.17 ^B^	2.17 ± 0.05 ^B^	2.05 ± 0.05 ^B^
8	α-Phellandrene	1001	1008	0.30 ± 0.04				
9	α-terpinene	1021	1018		0.18 ± 0.03	0.24 ± 0.02	0.19 ± 0.00	0.16 ± 0.01
10	Limonene	1034	1033	0.75 ± 0.07 ^A^	0.41 ± 0.03 ^B^	0.44 ± 0.03 ^B^	0.32 ± 0.01 ^B^	0.24 ± 0.02 ^B^
11	1,8-Cineole	1040	1039	16.70 ± 1.62 ^A^	35.70 ± 1.49 ^B^	38.70 ± 0.49 ^B^	37.90 ± 0.92 ^B^	33.21 ± 0.15 ^B^
12	γ-Terpinene	1063	1064	0.46 ± 0.04 ^A^	1.01 ± 0.04 ^B^	1.02 ± 0.06 ^B^	0.62 ± 0.02 ^A^	0.54 ± 0.03 ^A^
13	cis-Sabinene hydrate	1074	1076	0.20 ± 0.02 ^A^	0.18 ± 0.01 ^A^	0.17 ± 0.01 ^A^	0.18 ± 0.01 ^A^	0.18 ± 0.01 ^A^
14	Terpinolene	1094	1089		0.22 ± 0.00	0.27 ± 0.02	0.20 ± 0.01	0.14 ± 0.02
15	Linalool	1104	1104	0.85 ± 0.06 ^A^	0.68 ± 0.03 ^A^	0.73 ± 0.11 ^A^	0.87 ± 0.03 ^A^	1.03 ± 0.06 ^A^
16	α-Thujone	1112	1117	0.69 ± 0.05 ^A^	2.20 ± 0.04 ^B^	1.32 ± 0.03 ^B^	1.83 ± 0.05 ^B^	2.12 ± 0.03 ^B^
17	β-Thujone	1124	1127	0.99 ± 0.06 ^A^	1.51 ± 0.03 ^B^	1.21 ± 0.02 ^A^	1.56 ± 0.03 ^B^	1.54 ± 0.05 ^B^
18	Camphor	1155	1150	8.32 ± 0.53 ^A^	7.56 ± 0.08 ^A^	10.71 ± 0.15 ^B^	11.50 ± 0.24 ^B^	12.09 ± 0.06 ^B^
19	Borneol	1170	1170	0.66 ± 0.04 ^A^	0.60 ± 0.01 ^A^	0.90 ± 0.02 ^A^	0.22 ± 0.00 ^A^	0.85 ± 0.02 ^A^
20	Тerpinen-4-ol	1177	1178	3.50 ± 0.13 ^A^	3.13 ± 0.06 ^A^	2.71 ± 0.05 ^A^	3.66 ± 0.05 ^A^	3.42 ± 0.07 ^A^
21	*p*-Cymene-8-ol	1185	1183	1.02 ± 0.05 ^A^	0.31 ± 0.01 ^B^	0.52 ± 0.01 ^B^	0.19 ± 0.01 ^B^	0.65 ± 0.01 ^B^
22	Myrtenol	1188	1194	2.13 ± 0.09 ^A^	1.88 ± 0.05 ^B^	1.65 ± 0.01 ^B^	1.62 ± 0.02 ^B^	2.13 ± 0.04 ^A^
23	α-Terpineol	1204	1199	5.53 ± 0.15 ^A^	7.64 ± 0.22 ^A^	4.67 ± 0.02 ^A^	5.96 ± 0.07 ^A^	6.03 ± 0.07 ^A^
24	Neral	1236	1238	0.17 ± 0.01				
25	Carvone	1257	1258	0.13 ± 0.01 ^A^	0.06 ± 0.02 ^A^	0.16 ± 0.01 ^A^	0.15 ± 0.00 ^A^	
26	Linalyl acetate	1261	1259	0.22 ± 0.03 ^A^	0.10 ± 0.02 ^A^			
27	Bornyl acetate	1293	1288	0.24 ± 0.04 ^A^	0.17 ± 0.02 ^A^	0.16 ± 0.02 ^A^	0.29 ± 0.00 ^A^	
28	Thymol	1305	1293		0.11 ± 0.01			
29	Carvacrol	1324	1309	0.23 ± 0.01 ^A^	0.11 ± 0.00 ^A^	0.18 ± 0.00 ^A^	0.15 ± 0.04 ^A^	
30	Dihydrocarveol acetate	1357	1347	4.42 ± 0.17 ^A^	1.82 ± 0.17 ^B^	3.12 ± 0.05 ^B^	3.21 ± 0.02 ^B^	2.67 ± 0.06 ^B^
31	Eugenol	1378	1359		0.23 ± 0.01			
32	β-Bourbonene	1385	1384	0.15 ± 0.02 ^A^	0.11 ± 0.03 ^A^	0.12 ± 0.01 ^A^	0.16 ± 0.03 ^A^	
33	α-Cadinene	1424		0.17 ± 0.03				
34	β-Caryophyllene	1439	1426	13.88 ± 0.63 ^A^	10.63 ± 0.54 ^B^	8.73 ± 0.17 ^B^	7.02 ± 0.04 ^B^	10.14 ± 0.10 ^B^
35	Aromadendrene	1444	1440		0.11 ± 0.01			
36	5-Oxobornyl acetate	1452	1484			0.10 ± 0.00		
37	α-Humulene ne	1456	1456	0.75 ± 0.04 ^A^	0.77 ± 0.08 ^A^	0.86 ± 0.02 ^A^	0.74 ± 0.02 ^A^	0.81 ± 0.02 ^A^
38	*Allo*-Aromadendrene	1471	1462	4.58 ± 0.21 ^A^	1.80 ± 0.09 ^B^	2.04 ± 0.05 ^B^	1.52 ± 0.01 ^B^	2.99 ± 0.03 ^B^
39	γ-Muurolene	1478	1477		0.11 ± 0.01			
40	(z)-β-Guaiene	1489	1490	0.17 ± 0.04 ^A^	0.15 ^A^			
41	Viridiflorene	1497	1494	0.19 ± 0.02 ^A^	0.07 ± 0.01 ^B^			
42	γ-Cadinene	1513	1513	1.89 ± 0.10 ^A^	0.84 ± 0.05 ^B^	0.68 ± 0.02 ^B^	0.57 ± 0.01^B^	0.64 ± 0.02 ^B^
43	(Z)-Calamenene	1529	1526	0.30 ± 0.04 ^A^	0.08 ± 0.00 ^B^	0.13 ± 0.03 ^B^	0.13 ± 0.01 ^B^	0.15 ± 0.01 ^B^
44	δ-Cadinene	1537	1531	2.74 ± 0.24 ^A^	0.36 ± 0.02 ^B^	0.45 ± 0.02 ^B^	0.47 ± 0.02 ^B^	0.72 ± 0.03 ^B^
45	UD	1543		1.00 ± 0.14				
46	Viridiflorol	1595	1590	0.59 ± 0.19				
47	Humulene epoxide I	1599	1596	1.43 ± 0.07 ^A^	0.63 ± 0.03 ^B^	0.56 ± 0.02 ^B^	0.60 ± 0.02 ^B^	0.66 ± 0.01 ^B^
48	Humulene epoxide II	1605	1600	2.96 ± 0.18 ^A^	1.28 ± 0.07 ^B^	1.01 ± 0.04 ^B^	1.71 ± 0.03 ^B^	1.59 ± 0.06 ^B^
49	Humulene epoxide III	1616	1615	7.62 ± 0.25 ^A^	2.54 ± 0.14 ^B^	1.94 ± 0.06 ^B^	3.70 ± 0.11 ^B^	3.46 ± 0.08 ^B^
50	muurola-4,10(14)-dien-1-b-ol	1632	1625	0.83 ± 0.11 ^A^	0.21 ± 0.01 ^B^	0.23 ± 0.02 ^B^	0.30 ± 0.01 ^B^	0.55 ± 0.02 ^B^
51	UD	1656		0.35 ± 0.04				
52	14-hydroxy-(Z)-caryophyllene	1660	1667	2.10 ± 0.16 ^A^	0.34 ± 0.12 ^B^	0.24 ± 0.02 ^B^	0.37 ± 0.10 ^B^	0.60 ± 0.03 ^B^
53	UD	1667		0.79 ± 0.08				
54	α-Bisabolol	1675	1683	3.43 ± 0.36 ^A^	0.27 ± 0.01 ^B^	0.22 ± 0.02 ^B^	0.24 ± 0.02 ^B^	0.43 ± 0.01 ^B^
55	UD	1693		2.09 ± 0.06 ^A^	0.53 ± 0.03 ^B^	0.37 ± 0.01 ^B^	0.61 ± 0.04 ^B^	0.62 ± 0.03 ^B^
Identified components				46	46	40	39	33
Total Yields %				98.3 ± 2.39	98.43 ± 1.82	99.37 ± 0.18	99.26 ± 0.67	97.14 ± 0.35
Non-oxygenated monoterpenes				3.22	13.79	14.93	12.0	8.06
Non-oxygenated sesquiterpenes				24.82	15.03	13.01	10.61	15.45
Oxygenated monoterpenes				45.57	63.36	66.66	68.96	65.74
Oxygenated sesquiterpenes				18.96	5.27	4.2	6.92	7.29
Phenolics constituents				0.23	0.45	0.18	0.15	0

KI^exp.^: Experimental Kovats retention index; KI^rep.^: Reported Kovats retention index; FH: Fresh herb; 1WDH: One-week dried herb; 2WDH: Two-week dried herb; 3WDH: Three-week dried herb; 4WDH: Four-week dried herb; UD: Unidentified. The mean of 3 independent runs was used to calculate areas under the peaks. Tukey’s multiple comparisons were performed for the statistically significant constituents, using a one-way ANOVA. Different superscript letters (A–B) within the corresponding column show significant differences in the constituents’ percentage (*p* < 0.05) in the sage essential oil obtained from dried herb compared to the fresh herb.

**Table 3:** The effects of sage’s essential oils on liver functions in the AAP-induced liver toxicity in rats as compared with the control and silymarin groups.

Test	AST IU/L	ALT IU/L	ALP IU/L	Total Protein gm/dL
Control group	93.32 ± 41.39	28.82 ± 2.731	111.3 ± 11.11	7.903 ± 0.28
AAP group	202.6 ± 36.60 ^a^	59.56 ± 21.55 ^a^	260.5 ± 40.72 ^a^	4.133 ± 0.195 ^a^
AAP + FH	132.2 ± 14.95 ^b^	46.55 ± 17.25 ^b^	103.0 ± 4.29 ^b^	8.597 ± 0.22 ^b^
AAP + 1WDH	123.9 ± 6.671 ^b^	25.89 ± 18.27 ^b^	138.2 ± 15.32 ^b^	8.670 ± 0.81 ^b^
AAP + 2WDH	122.4 ± 15.13 ^b^	23.75 ± 3.064 ^b^	144.6 ± 19.29 ^b^	8.810 ± 0.61 ^b^
AAP + 3WDH	139.2 ± 31.64 ^b^	25.89 ± 3.397 ^b^	102.0 ± 1.08 ^b^	9.210 ± 0.20 ^b,c^
AAP + 4WDH	141.9 ± 18.35 ^b^	20.40 ± 5.143 ^b,d^	114.4 ± 8.46 ^b^	9.035 ± 0.25 ^b,c^
AAP + silymarin	132.4 ± 23.30 ^b^	36.10 ± 9.336 ^b^	118.7 ± 9.90 ^b^	7.910 ± 0.25 ^b^
*p*-value	0.001 **	0.001 **	0.001 **	0.001 **

AAP, acetaminophen (paracetamol); FH, essential oil obtained from a fresh sample of sage; 1WDH, 2WDH, 3WDH, and 4WDH referred to the essential oil obtained from sage herb sample dried for one week, two weeks, three weeks, and four weeks, respectively. The results are expressed as mean ± SD; a = Significant difference, compared to controls, b = Significant difference compared to AAP group, c = Significant difference compared to silymarin group, d = Significant difference compared to fresh extract of sage, *p*-value is significant if it ≤ 0.05. ** is indicated for *p*-value < 0.001.

**Table 4:** The prophylactic effect of sage essential oil on blood cholesterol, triglycerides, urea, and creatinine in acetaminophen-induced liver toxicity in rats as compared with the control and silymarin groups.

Test (Unit/L)	Cholesterol mg/dL	Triglycerides mg/dL	Urea mg/dL	Creatinine mg/dL
Control group	86.87 ± 10.37	137.4 ± 2.117	34.28 ± 8.15	0.62 ± 0.33
AAP group	119.3 ± 37.25 ^a^	160.1 ± 18.81 ^a^	52.28 ± 6.61 ^a^	1.43 ± 0.39 ^a^
AAP + FH	73.20 ± 8.39 ^b^	130.3 ± 2.20 ^b^	23.23 ± 17.57 ^b^	0.92 ± 0.06 ^b^
AAP + 1WDH	84.66 ± 6.99 ^b^	141.8 ± 7.91 ^b^	42.66 ± 20.69	0.76 ± 0.21 ^b^
AAP + 2WDH	85.89 ± 10.45 ^b^	162.0 ± 1.69	47.75 ± 5.383	0.54 ± 0.37 ^b^
AAP + 3WDH	84.10 ± 14.47 ^b^	137.1 ± 7.12 ^b^	41.29 ± 12.38	0.86 ± 0.16 ^b^
AAP + 4WDH	79.13 ± 11.53 ^b^	138.3 ± 7.42 ^b^	39.46 ± 11.00	0.91 ± 0.08 ^b^
AAP + silymarin	75.45 ± 6.54 ^b^	152.0 ± 19.56 ^b^	46.13 ± 19.91	0.89 ± 0.09 ^b^
*p*-value	0.001 ***	0.001***	0.01 **	0.001 ***

AAP, acetaminophen (paracetamol); FH, essential oil obtained from a fresh sample of sage; 1WDH, 2WDH, 3WDH, and 4WDH referred to the essential oil obtained from sage herb sample dried for one week, two weeks, three weeks, and four weeks, respectively. The results are expressed as mean ± SD, a = Significant difference compared to control group, b = Significant difference compared to AAP group. ** and *** are indicated for *p*-value < 0.01 and *p*-value < 0.001, respectively.

**Table 5:** IC_50_ and selective index values obtained for fresh and dried essential oil batches of sage against different cancer and normal fibroblast lung (MRC-5) cell lines.

Groups	MCF-7		HepG-2		HeLa		MRC-5
	IC_50_ ± SD	SI	IC_50_ ± SD	SI	IC_50_ ± SD	SI	IC_50_ ± SD
DOX	1.4 ± 0.1	1.1	1.04 ± 0.1	1.5	1.02 ± 0.1	1.6	1.6 ± 0.1
FH	181.3 ± 18.3	3.3	161.7 ± 15.3	3.7	142.1 ± 12.4	4.2	596.5 ± 20
1WDH	189.3 ± 17.45	2.2	179.12 ± 16.5	2.4	164.1 ± 15.5	2,6	425.6 ± 30.2
2WDH	194.1 ± 20.1	2.6	182.4 ± 14.2	2.7	174.7 ± 13.6	2.9	499.4 ± 32
3WDH	176.3 ± 16.5	2.3	174.9 ± 13.2	2.3	127.5 ± 11.4	3.2	405.2 ± 18.3
4WDH	215.7 ± 18.4	1.9	195.4 ± 20	2.1	176.2 ± 18	2.4	414.7 ± 27.1

## Data Availability

Data are provided in the main text and supplementary file.

## Supplementary Materials

The following are available online at, Excel sheet of the GC-FID analysis raw data.
